# The Genomic Impact of Mycoheterotrophy in Orchids

**DOI:** 10.3389/fpls.2021.632033

**Published:** 2021-06-09

**Authors:** Marcin Jąkalski, Julita Minasiewicz, José Caius, Michał May, Marc-André Selosse, Etienne Delannoy

**Affiliations:** ^1^Department of Plant Taxonomy and Nature Conservation, Faculty of Biology, University of Gdańsk, Gdańsk, Poland; ^2^Institute of Plant Sciences Paris-Saclay, Université Paris-Saclay, CNRS, INRAE, Univ Evry, Orsay, France; ^3^Université de Paris, CNRS, INRAE, Institute of Plant Sciences Paris-Saclay, Orsay, France; ^4^Sorbonne Université, CNRS, EPHE, Muséum National d’Histoire Naturelle, Institut de Systématique, Evolution, Biodiversité, Paris, France

**Keywords:** mycorrhiza, photosynthesis, metabolic evolution, mycoheterotrophy, orchids, transcriptome, *Epipogium aphyllum*, *Neottia nidus-avis*

## Abstract

Mycoheterotrophic plants have lost the ability to photosynthesize and obtain essential mineral and organic nutrients from associated soil fungi. Despite involving radical changes in life history traits and ecological requirements, the transition from autotrophy to mycoheterotrophy has occurred independently in many major lineages of land plants, most frequently in Orchidaceae. Yet the molecular mechanisms underlying this shift are still poorly understood. A comparison of the transcriptomes of *Epipogium aphyllum* and *Neottia nidus-avis*, two completely mycoheterotrophic orchids, to other autotrophic and mycoheterotrophic orchids showed the unexpected retention of several genes associated with photosynthetic activities. In addition to these selected retentions, the analysis of their expression profiles showed that many orthologs had inverted underground/aboveground expression ratios compared to autotrophic species. Fatty acid and amino acid biosynthesis as well as primary cell wall metabolism were among the pathways most impacted by this expression reprogramming. Our study suggests that the shift in nutritional mode from autotrophy to mycoheterotrophy remodeled the architecture of the plant metabolism but was associated primarily with function losses rather than metabolic innovations.

## Introduction

More than 85% of vascular plants grow in association with soil fungi, forming a mycorrhizal symbiosis (Wang and Qiu, 2006; van der Heijden et al., 2015; Brundrett and Tedersoo, 2018). Thanks to this symbiosis, plant growth, and fitness are substantially improved by better mineral nutrition and increased resistance to biotic and abiotic stresses (see Jung et al., 2012 for a review). In this mutualism, the fungal partner provides mineral nutrients (e.g., water, N, P, and K) in exchange for organic compounds from the photosynthesis of the plant (see Rich et al., 2017 for a review). However, more than 500 plant species, called mycoheterotrophs, have lost their ability to photosynthesize and entirely rely on their fungal partners for mineral and organic nutrients, reversing the usual direction of the net carbon flow. The metabolic evolution associated with this switch, which is still poorly understood, has occurred in parallel at least 50 times in 17 plant families, including at least 30 independent transitions in Orchidaceae (Merckx et al., 2009; Merckx and Freudenstein, 2010; Tĕšitel et al., 2018; Barrett et al., 2019). One of the characteristics that may make orchids more prone to this evolutionary change in their nutrition mode lies in their minute seeds devoid of nutritional reserves (Rasmussen, 1995). The seeds fully depend on their mycorrhizal fungi for carbon supply at germination and early developmental stages (protocorms), which are thus always mycoheterotrophic (Merckx, 2013; Dearnaley et al., 2016). Most orchid species later shift to autotrophy once photosynthesis becomes possible and establish more reciprocal exchanges with mycorrhizal fungi at adulthood. However, some species still recover carbon from the fungi at adulthood in addition to their photosynthesis (mixotrophy; Selosse and Roy, 2009), and from this nutrition some even evolved complete loss of photosynthesis and mycoheterotrophy at adulthood (Merckx et al., 2009; Dearnaley et al., 2016). This versatile relation between orchids and their mycorrhizal partners provides an useful framework for understanding the metabolic evolution resulting in mycoheterotrophy (Suetsugu et al., 2017; Lallemand et al., 2019b).

The impact of mycoheterotrophy on plant physiology can be analyzed through the changes in genomes of mycoheterotrophs compared to autotrophic relatives. As mycoheterotrophy is associated with the loss of photosynthesis, sequencing of the plastid genome has been targeted first, thanks to next-generation methods (e.g., Logacheva et al., 2014; Schelkunov et al., 2015; Lim et al., 2016; Ravin et al., 2016; Yudina et al., 2021). A common feature among plastid genomes of mycoheterotrophs is a strong reduction in size and gene content, especially with, as expected, a loss of all photosynthetic genes (Graham et al., 2017; Hadariová et al., 2018). However, the plastid genome contains only a tiny fraction of plant genes and the absence of genes from the plastid genome does not rule out the possibility that some of them were transferred into the nuclear genome, rather than lost (Bock, 2017).

Furthermore, in addition to photosynthesis, the transition to mycoheterotrophy can be expected to affect other metabolic processes, which cannot be assessed without the complete gene repertoire of all three plant genomes. Out of three published full genomes of heterotrophic plants, two belong to obligate plant parasites (Vogel et al., 2018; Yoshida et al., 2019) and one to an east Asian mycoheterotrophic orchid (*Gastrodia elata* Blume; Yuan et al., 2018). When compared to photosynthetic orchids, the genome of *G. elata* is characterized by a reduction of gene content, including the loss of most of the genes associated with photosynthesis, and the reduction of gene families involved in resistance to pathogens. At the same time, it shows an expansion of gene families that are putatively involved in the interaction with fungi (Yuan et al., 2018).

Despite the decrease in sequencing costs, the *de novo* characterization of a complete plant genome is still = expensive and tedious, especially in the case of relatively large genomes of achlorophyllous orchids, [from about 6 Gb for *Corallorhiza trifida* Chatelain to about 16 Gb for *Neottia nidus-avis* (L.) L.C.M. Rich; Pellicer and Leitch, 2020]. Another approach for studying gene content is to analyze transcriptomes. RNA-seq focuses on the transcribed fraction of the genome, which includes the protein-coding genes. Transcriptomes of five mycoheterotrophic plants are currently available (Schelkunov et al., 2018; Leebens-Mack et al., 2019). The transcriptomes of two orchids, *Epipogium aphyllum* Sw. and *Epipogium roseum* (D. Don) Lindl., and the Ericaceae *Monotropa hypopitys* L. show a loss of the photosynthetic genes (Schelkunov et al., 2018). Surprisingly, but in accordance with results from obligate parasitic plants (Wickett et al., 2011; Chen et al., 2020), the chlorophyll synthesis pathway was mostly conserved in these plants, even if incomplete. However, transcriptome analysis only identifies the genes expressed in the tissue(s) under study, and as the previous studies of mycoheterotrophic species concentrated on the aerial part only, a fraction of the extant genes was likely missed. In addition, the missed genes include all the genes specifically expressed in the roots and mycorrhiza, which are fundamental to understanding of the mechanism of the interaction between a mycoheterotrophic plant and its fungal partners. Finally, it is the most likely that the switch to mycoheterotrophy not only results in gene losses, but also in neofunctionalizations and changes in the expression profiles of some retained genes, which are difficult or impossible to capture in simple analyses of gene repertoires.

**FIGURE 1:**
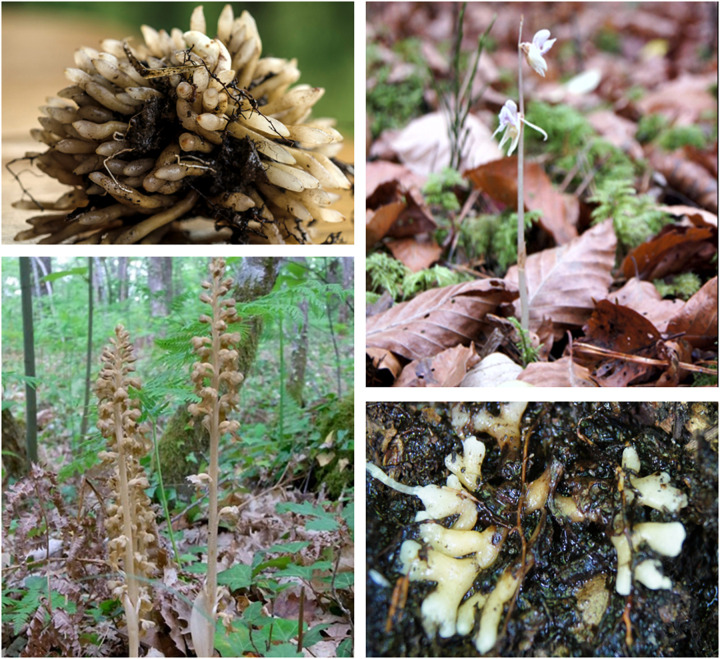
Morphology of *Neottia nidus-avis* and *Epipogium aphyllum*. Top left: roots of *N. nidus-avis*. Bottom left: inflorescence of *N. nidus-avis*. Top right: inflorescence of *E. aphyllum* (Courtesy of Emilia Krawczyk). Bottom right: rhizome of *E. aphyllum* (Courtesy of Emilia Krawczyk).

Here, we explored the transcriptome and gene expression profiles in the mycorrhiza, stems, and flowers of the MH orchids *N. nidus-avis* and *E. aphyllum* (Figure 1). Both studied species are achlorophyllous and, like *G. elata*, belong to the orchid subfamily Epidendroideae. Despite their rarity, they have a broad Eurasian range (Hulten and Fries, 1986) and, together with *G. elata*, they represent three independent evolutionary origins of mycoheterotrophy in orchids (Merckx and Freudenstein, 2010). Their shoots have minute achlorophyllous scales and produce a few large flowers in *E. aphyllum* (Taylor and Roberts, 2011) and many small flowers in *N. nidus-avis* (Selosse, 2003). Both species are considered allogamous, producing scent and a little amount of nectar (Ziegenspeck, 1936; Claessens, 2011; Jakubska-Busse et al., 2014; Krawczyk et al., 2016) however some level of autogamy is also expected in *N. nidus-avis* (Claessens, 2011).

Considering their underground parts, *N. nidus-avis* has a clump of short and thick mycorrhizal roots growing out of a short and thin rhizome, and *E. aphyllum* forms a fleshy, dichotomously branched and rootless rhizome that hosts the fungus (Roy et al., 2009). Thus, roots of *N. nidus-avis* and the rhizome of *E. aphyllum* are not anatomically equivalent but both correspond to the underground organs where the interactions with their fungal partner are taking place.

We used RNA-seq in flowers, stems, and mycorrhizal parts (roots or rhizomes) sampled in natural forest conditions, and identified expressed gene sets in each case. In combination with published data from the *G. elata* genome, we compared the gene sets of the three mycoheterotrophic orchids to that of three autotrophic orchid species, in order to highlight the gene losses and gains associated with the switch to mycoheterotrophy in orchids. We also identified genes that are differentially expressed between the three investigated tissues. As no equivalent dataset (expression profiles per organ for the same individuals with biological replicates) was available for autotrophic orchids at the time of study, we compared these profiles to expression in other autotrophic non-orchid plants. This comparison suggested that, in addition to gene losses, the switch to mycoheterotrophy induced extensive expression reprogramming.

## Results

### Sequencing, *de novo* Assembly and Functional Annotation

In total, 12 cDNA libraries from flowers, stems, and mycorrhizal roots (two replicates per tissue and species) were sequenced resulting in 304,280,061 reads for *N. nidus-avis* and 178,486,849 reads for *E. aphyllum*. After assembly and filtering of the probable contaminating transcripts, the final set of transcripts comprised 44,451 and 38,488 sequences for *N. nidus-avis* and *E. aphyllum*, respectively (Table 1). As expected, the fraction of contaminating contigs was much higher in the mycorrhizal samples (roots in *N. nidus-avis* and rhizome in *E. aphyllum*), and indeed almost all the contaminating transcripts were most probably of fungal origin (Supplementary Tables 3, 4). Thanks to the presence of a few contigs corresponding to ITS, the main fungal partners can be identified as *Inocybe cervicolor* (Pers.) Quél. and *Hebeloma incarnatulum* A.H. Sm. for *E. aphyllum* and *Sebacina epigaea* (Berk. & Broome) Bourdot & Galzin for *N. nidus-avis* as expected (McKendrick et al., 2002; Selosse et al., 2002; Roy et al., 2009).

**TABLE 1:** Statistics of the final assemblies.

	*Neottia nidus-avis*	*Epipogium aphyllum*
Number of transcripts	43,451	38,488
Number of “genes”	39,731	36,275
Median/mean transcript length (bp)	675/993	555/920
Shortest/longest transcript (bp)	201/13,537	201/15,415
Transcripts over 1/10k bp length	15,391/10	11,980/9
Transcript n50 (bp)	1,506	1,480
GC %	45.56	44.26
Total assembled bases	43,160,528	35,412,792

We functionally annotated the transcripts recovered for the two studied species. Roughly 46 and 50% of the transcripts could be attributed to any annotation category in *N. nidus-avis* and *E. aphyllum*, respectively (Supplementary Table 5 and Supplementary Data 1).

We also assessed the completeness of the generated transcriptomes using several analyses. The BUSCO (Benchmarking Universal Single-Copy Orthologs) analysis showed 78.5 and 71% of completeness for *N. nidus-avis* and *E. aphyllum*, respectively, comparable to or higher than that for the *G. elata* genome (73.1%), and much higher than that for the *E. aphyllum* transcriptome generated by Schelkunov et al. (2018) (53.4%; Supplementary Table 6). We also checked the mapping rate of the RNA-seq reads on these transcriptomes (Supplementary Table 7). This was higher than 94%, except for mycorrhizal samples as expected due to the presence of the mycorrhizal fungi. Finally, we looked for the orthologs of the plant KEGG pathways and of the Mapman4 bins and compared them to the *G. elata* gene content (Supplementary Data 2). An ortholog was counted if at least one transcript or gene was associated with it. Out of the 140 tested KEGG pathways representing 15,150 potential orthologs, none were differentially represented between our transcriptomes and *G. elata*. Similarly, none of the 1,196 tested Mapman4 bins representing 4,966 potential orthologs were differentially represented. Even when relaxing the stringency of the test (raw *p*-value < 0.05), no bin or pathway suggesting missed orthologs in our transcriptomes compared to *G. elata* was statistically significant. Taken together, these results strongly support that our transcriptomes were complete at least for the pathways considered in the comparison and that any missing orthologs from our transcriptomes were probably lost by the corresponding species. It also suggests that the gene repertoires of *E. aphyllum*, *N. nidus-avis*, and *G. elata* are very similar.

### Impact of Mycoheterotrophy on Gene Repertoires in Mycoheterotrophic Orchids

By considering the gene repertoires of three mycoheterotrophic orchids that experienced independent evolutionary origins of mycoheterotrophy, we can also address its impact on their gene sets. A comparison with the genomes of *Phalaenopsis equestris*, *Dendrobium catenatum*, and *Apostasia shenzhenica*, three autotrophic orchids, using the KEGG and Mapman4 pathways described above (Table 2 and Supplementary Data 2), shows that the switches to mycoheterotrophy result in the loss of orthologs exclusively associated with pathways directly related to photosynthesis. Even when relaxing the stringency of the test (raw *p*-value < 0.05), there is no indication that the switch to mycoheterotrophy is associated with any gain of novel genes or that pathways other than those associated with plastid or photosynthesis showed significant gene losses (Supplementary Data 2). It should also be noted that none of the genes lost from their plastid genomes were found in the transcriptomes of *E. aphyllum* and *N. nidus-avis*. This suggests that the switch to mycoheterotrophy selectively resulted in gene losses (and not in gene transfers to their nucleus) in pathways associated with photosynthesis. However, most of these pathways were not completely lost.

**TABLE 2:** Gene content: pathways impacted by the switch to mycoheterotrophy.

	Code	Size	AS	DC	PE	GE	EA	NNA	MH impact
**Mapman4**									
Photosynthesis	1	291	161	196	183	42	33	41	Losses
Photophosphorylation	1.1	239	124	157	143	17	13	18	Losses
ATP synthase complex	1.1.9	12	6	12	12	0	0	1	Losses
Chlororespiration	1.1.8	41	22	17	4	5	4	4	Losses
Cytochrome b6/f complex	1.1.2	19	10	19	19	0	0	0	Losses
Linear electron flow	1.1.5	5	4	5	5	1	1	1	Losses
Photosystem I	1.1.4	28	20	26	26	1	1	0	Losses
Photosystem II	1.1.1	74	58	74	73	10	7	12	Losses
Calvin cycle	1.2	30	22	24	25	12	7	10	Losses
RuBisCo activity	1.2.1	14	13	13	14	7	4	4	Losses
Galactolipid and sulfolipid biosynthesis	5.3	7	7	7	7	3	3	4	Losses
Coenzyme metabolism	7	224	145	155	154	129	132	135	Losses
Phylloquinone biosynthesis	7.13	8	8	8	8	2	2	2	Losses
Tetrapyrrole biosynthesis	7.12	58	37	39	38	25	27	29	Losses
Chlorophyll metabolism	7.12.6	23	21	22	22	10	11	12	Losses
Organelle RNA polymerase machinery	15.6	35	21	28	29	7	6	6	Losses
Organelle RNA polymerase activities	15.6.1	27	19	26	27	6	5	5	Losses
Organelle RNA processing	16.12	102	73	79	72	55	49	53	Losses
Organelle RNA editing	16.12.5	42	33	36	28	23	17	19	Losses
Organelle RNA stability	16.12.4	6	6	6	5	3	2	2	Losses
Chloroplast disulfide bond formation	18.11.2	3	3	3	3	0	0	1	Losses
Plastid movement	20.5	10	9	9	9	4	4	4	Losses
Total	total	5963	3945	4211	4185	3790	3813	3891	Losses
**KEGG pathways**									
Photosynthesis	195	63	54	54	33	10	4	4	Losses
Photosynthesis – antenna proteins	196	42	11	11	11	0	3	1	Losses

*Code, code of the Mapman4 bin or KEGG pathway; Size, ortholog content of the bin or pathway; AS, A. shenzhenica; DC, D. catenatum; PE, P. equestris; GE, G. elata; EA, E. aphyllum; NNA, N. nidus-avis; MH impact, impact of the switch to mycoheterotrophy.*

**FIGURE 2:**
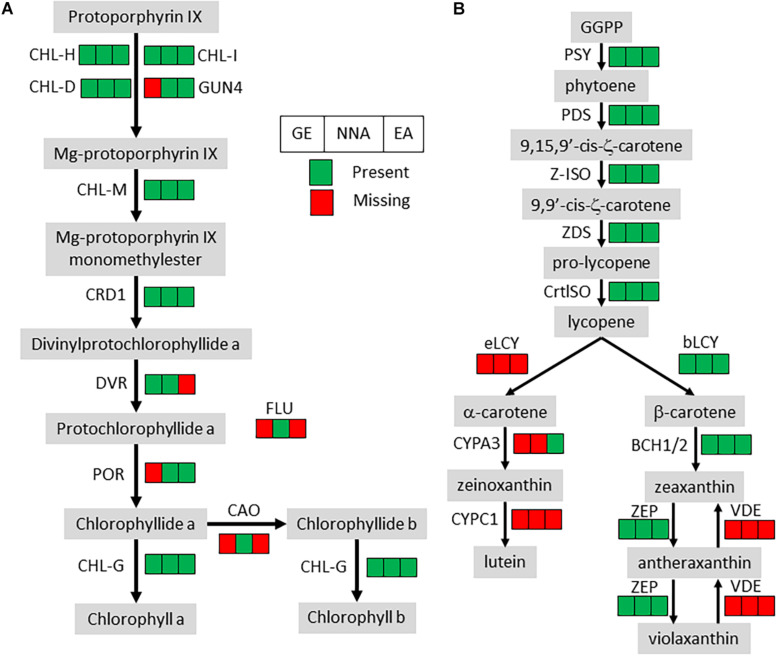
Pigment synthesis pathways in mycoheterotrophic orchids. GE, *G. elata*; NNA, *N. nidus-avis*; EA, E. *aphyllum*; **(A)** Chlorophyll biosynthesis. CHL-D, CHL-H, CHL-I, GUN4, magnesium chelatase; CHL-M, Mg-protoporphyrin IX O-methyltransferase; CRD1, Mg-protoporphyrin IX monomethylester cyclase; DVR, divinyl chlorophyllide-a 8-vinyl-reductase; POR, protochlorophyllide oxidoreductase; FLU, glutamyl-tRNA reductase regulator; CAO, chlorophyllide a oxygenase; CHL-G, chlorophyll synthase. **(B)** Carotenoid biosynthesis. PSY, phytoene synthase; PDS, phytoene desaturase; Z-ISO, ζ-carotene isomerase; ZDS, ζ-carotene desaturase; CrtISO, carotenoid isomerase; eLCY, lycopene ε-cyclase; bLCY, lycopene β-cyclase; BCH1/2, β-ring carotene hydroxylase; ZEP, zeaxanthin epoxidase; VDE, violaxanthin de-epoxidase; CYPA3, α-carotene β-ring hydroxylase; CYPC1, carotenoid ε-hydroxylase.

All the orthologs required for photosystems were not detected, but the losses in the chlorophyll metabolism pathway were almost exclusively restricted to chlorophyll degradation and interconversion. As seen before (Wickett et al., 2011; Schelkunov et al., 2018), the chlorophyll synthesis pathway was mostly conserved but incomplete in *G. elata* and *E. aphyllum* (Figure 2A). By contrast, *N. nidus-avis* expressed the full extent of genes required for the biosynthesis of chlorophyll as well as some chlorophyll *a/b* binding proteins [Light-Harvesting-Complex A3 (LHCA3), LHCB1, LHCB2, stress-enhanced protein 1 (SEP1), SEP3, SEP5, and early light-induced protein (ELIP) genes]. Similarly, the three mycoheterotrophic species were missing the *lycE* and *lut5* genes required for the synthesis of lutein, a photoprotective pigment, but possessed a complete biosynthesis pathway to violaxanthin (Figure 2B). No gene coding for a violaxanthin de-epoxidase, required for the xanthophyll cycle to occur, was found in any of the three MH species.

Gene losses in MH species mirror the loss of photosynthesis but another characteristic of MH species is the lack of developed leaves which are reduced to small scales. Leaf initiation and expansion is controlled by a network of transcription factors and hormone gradients (Bar and Ori, 2014; Wang et al., 2021). None of the important gene families involved in leaf development and present in the genome of autotrophic orchids (YABBY, KNOX, BOP, PINOID, KNAT, ARF, HD-ZIPIII, and NGATHA) are missing from MH orchids.

An examination of known pathways, mainly deriving from model autotrophic plants, allows identification of gene losses linked to the switch to mycoheterotrophy, but this misses any potential new pathways or genes that may be associated with this transition. To address this, we performed an orthology analysis including the coding genes of the six previous orchid species and three grasses [*Zea mays*, *Brachypodium distachyon*, and *Oryza sativa* (Supplementary Data 3 and Supplementary Tables 8, 9)]. Out of the 18,259 orthogroups identified, only 38 contained exclusively genes from all three MH orchid species. Twenty-two of these orthogroups contained only unannotated genes and the 16 remaining did not have specific annotations (Supplementary Data 4). These results suggest that the switch to mycoheterotrophy in orchids does not involve new pathways or functions.

### A Transcriptome Analysis Highlights the Organ-Specific Functions of Mycoheterotrophic Orchids

The pairwise comparisons of the transcriptome profiles of flower, stem, and mycorrhizal root/rhizome of *E. aphyllum* and *N. nidus-avis* identified the genes differentially expressed between these organs as well as organ-specific genes (Supplementary Data 5). We identified 18,817 and 12,331 differentially expressed genes as well as 6,351 and 4,520 organ-specific genes in *N. nidus-avis* and *E. aphyllum*, respectively (Table 3). The highest numbers of differentially expressed genes were observed between underground and aerial organs. Similarly, most organ-specific genes were identified in the mycorrhizal root/rhizome.

**TABLE 3:** Summary of differential gene expression analyses among the sampled tissues.

	*Neottia nidus-avis*	*Epipogium aphyllum*
Stem vs. flower	9,109/4,644 down, 4,465 up	5,315/2,123 down, 3,192 up
Mycorrhiza vs. flower	13,701/6,465 down, 7,236 up	7,596/3,430 down, 4,166 up
Mycorrhiza vs. stem	11,360/4,866 down, 6,494 up	7,849/3,955 down, 3,894 up
Flower-specific	55	297
Stem-specific	508	175
Mycorrhiza-specific	5,788	4,048
Total	25,168 (57.92%)	16,851 (43.78%)

**FIGURE 3:**
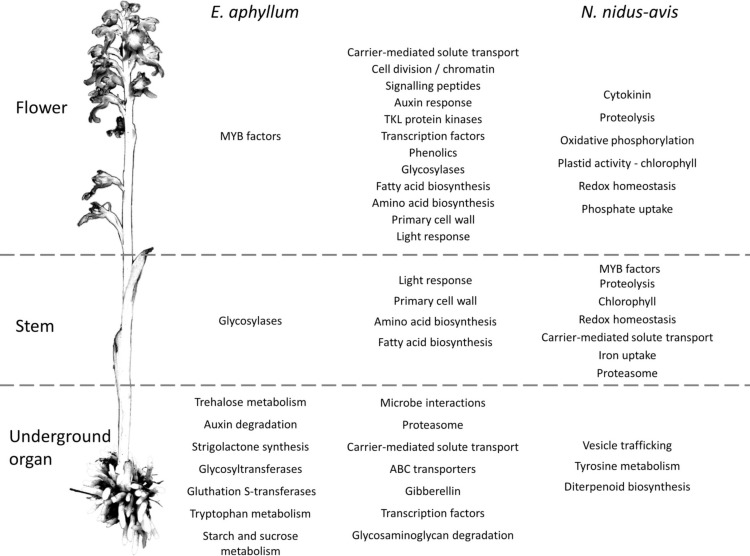
Pathways differentially expressed between organs in *E. aphyllum* and *N. nidus-avis*. Summary of the enrichment analysis of the transcriptomic expression profiles (Supplementary Data 5). A pathway is indicated in the organ(s) where its activity peaks. The common changes are shown in the central column while the changes specific to *E. aphyllum* are shown in the left column and those specific to *N. nidus-avis* in the right column. The terms are mostly derived from the Mapman4 and KEGG pathways.

To elucidate which functions are served by the differentially expressed and organ-specific genes, Gene Ontology, Mapman, and KEGG enrichment analyses were performed (Supplementary Data 6). While very few enrichments were found in the organ-specific genes, the differentially expressed genes showed that numerous metabolic functions were differentially activated in the three organs, following a strikingly similar pattern in *N. nidus-avis* and *E. aphyllum*. Figure 3 summarizes the Mapman and KEGG enrichment analyses, which are fully supported by the GO enrichment analyses. The metabolic functions are indicated where their transcriptomic activity appeared to be peaking. The aerial parts shared high levels of amino acid and fatty acid synthesis as well as high level of primary cell wall metabolism. Light signaling pathways were also activated in aerial parts. The flowers specifically showed high cell division and phenolic activities. In *N. nidus-avis*, the chlorophyll synthesis pathway was active mostly in the flowers along with other plastid associated pathways. At the other end of the plant, the mycorrhizal roots of *N. nidus-avis* and the mycorrhizal rhizomes of *E. aphyllum* showed some contrasted metabolic functions. Nevertheless they also showed a remarkable convergence with an increased activity of pathways related to pathogen and symbiont interactions, as well as of the transportome (e.g., ABC transporters and solute carriers) and degradation capacities (i.e., proteasome and glycosaminoglycan degradations).

### Comparison of Expression Profiles in Underground Organ and Stem of Mycoheterotrophic and Autotrophic Species

To understand the consequences of mycoheterotrophy for the expression profiles, it would be preferable to compare our mycoheterotrophic orchids to autotrophic relatives species from a transcriptomic point of view. However, no equivalent transcriptomic dataset (pairs of underground and stem organs from the same individuals with biological replicates) for autotrophic orchids or other monocots was publicly available at the time of analysis. We used datasets from two grasses, *B. distachyon* and *Z. mays*. We analyzed only the 8,620 (out of 18,259) orthogroups detected in the roots or stem of all four species. This filter removes most of the orthogroups associated with photosynthesis, but these pathways are an obvious major difference between the two trophic types. While 2,378 and 3,617 orthogroups were differentially expressed between underground organ and stem in autotrophic and mycoheterotrophic species, respectively, 3,359 orthogroups (39% of the analyzed orthogroups) showed a significantly different underground to aboveground ratio between the two trophic types, including 2,536 (30%) with inverted ratios (Supplementary Data 7).

**FIGURE 4:**
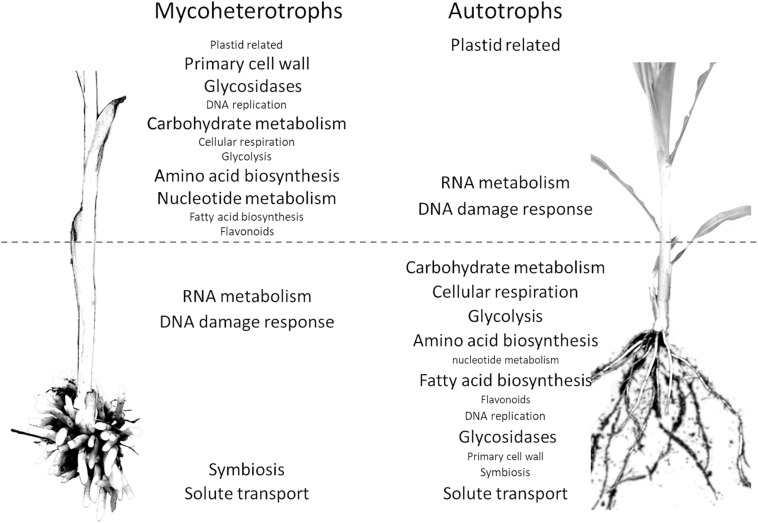
Comparison of distribution of pathways between the organs of mycoheterotrophic orchids and autotrophic non-orchid plants. Pathways enriched in the orthogroups showing a significantly different underground organ/stem expression ratio between mycoheterotrophic species (*N. nidus-avis* and *E. aphyllum*) and autotrophic species (*B. distachyon* and *Z. mays*). The pathways are indicated in the organ where their expression is highest. The pathways shown with a large font are also differentially expressed between underground organ and stem.

The pathway enrichment analysis of the differentially expressed orthogroups in the mycoheterotrophic orchids (Supplementary Data 8) showed results similar to the transcriptomic analysis of *E. aphyllum* and *N. nidus-avis* genes, supporting the idea that orthogroup expression patterns are biologically relevant. Figure 4 summarizes the results of the pathway enrichment analysis of these orthogroups. It is particularly noteworthy that the orthogroups associated with fatty acid biosynthesis, amino acid biosynthesis, primary cell wall metabolism, glycosidases and secondary metabolism are more expressed in the stem of the mycoheterotrophic orchids than in their underground organs and are more expressed in the roots of autotrophic grasses than in their stem. The opposite is true for the orthogroups involved in RNA metabolism and DNA damage response. The orthogroups of some pathways (those involved in solute transport, symbiosis, trehalose degradation, and cytochrome P450) were more expressed in the underground organs than in the stems for both autotrophic and mycoheterotrophic species but differed between the two suggesting that the species of the two trophic types either induced these pathways to different levels or used different orthologs.

The latter can be illustrated for the solute transport pathway. The 192 orthogroups showing a different root/shoot ratio between AT and MH (out of 392 orthogroups belonging to the solute transport pathway) are distributed in most transporter families, and in each family there are orthologs showing different behavior in MH and AT (Supplementary Data 7). This collectively illustrates that the consequences of mycoheterotrophy extend well beyond photosynthesis and associated gene losses observed previously. Mycoheterotrophy has remodeled a large fraction of gene expression and metabolism.

## Discussion

### No Genetic Innovation, but Convergent Gene Loss in Mycoheterotrophic Orchids

Using the RNA-seq data from rhizome/root, stem and flowers of *E. aphyllum* and *N. nidus-avis*, we identified gene sets that are probably almost complete, based on their comparison with the genome of *G. elata* (Supplementary Data 2). This comparison also illustrates that these three independent evolutionary events of mycoheterotrophy have converged to essentially the same gene repertoire. When comparing the molecular functions encoded in these three mycoheterotrophic orchids with those of autotrophic orchids, the switch to mycoheterotrophy entailed a global reduction of molecular functions, as was previously demonstrated for *G. elata* (Supplementary Data 2; Yuan et al., 2018). In addition, we could not detect any major gain of function associated with mycoheterotrophy. Obviously, it is difficult to identify potentially unknown functions and our transcriptome analysis must have missed some genes, potentially including those specifically expressed during germination. However, all orchids regardless of their nutrition at adulthood, are mycoheterotrophic during germination (Bernard, 1899; Merckx, 2013; Dearnaley et al., 2016). This indirectly indicates that they have all the genes and metabolic pathways required to obtain nutrients through mycoheterotrophic nutrition, suggesting that major gains/innovation are not essential for the transition to mycoheterotrophy. When looking for orthologs present in our three mycoheterotrophic orchid species, but not in the other six autotrophic species, we found only a handful of mycoheterotrophy-specific orthologs. It is highly unlikely that they are the key genes required for the switch to mycoheterotrophy, although more extensive sampling of mycoheterotrophic and autotrophic species should be done to verify this assumption.

In addition to a general reduction of gene content, Yuan et al. (2018) showed that some gene families, mostly associated with interactions with fungi, expanded in the *G. elata* genome. Our transcriptome assemblies include large numbers of contigs putatively coding for enzymes such as mannose-specific lectins or β-glucosidases, indicating the possible expansion of some gene families in *E. aphyllum* and *N. nidus-avis*. However, using transcriptome assemblies (and despite or because of a step of redundancy reduction in our analysis), it is difficult to count the number of genes precisely because it is not possible to distinguish between two transcript isoforms and two copies of a gene. Only high-quality assemblies of the large genome of these species (16.96 Gb for *N. nidus-avis*; Vesely et al., 2012) will allow the confirmation of the expansion of such gene families in these species.

### Pigments and Secondary Metabolism: Compensatory Protection and Camouflage?

The gene losses observed in the mycoheterotrophic orchids reflect the evolution of their plastomes: massive gene loss restricted to photosynthetic pathways and functions. The only genes retained in their plastid genomes have non-photosynthetic functions (Graham et al., 2017; Barrett et al., 2019; Mohanta et al., 2020). By extension to the nuclear genome, we can assume that the orthologs not detected in mycoheterotrophic species are probably exclusively associated with photosynthesis, while the conserved orthologs probably have non-photosynthetic functions. Thus, the comparison of the gene contents of mycoheterotrophic and autotrophic species should provide useful information for the functional analysis of genes even in model plants, as shown by two examples below.

The loss of photosynthesis resulted in gene losses in several pigment synthesis pathways (Table 2). In *N. nidus-avis*, Pfeifhofer (1989) detected high amounts of zeaxanthin but no lutein. In the three MH species, the genes coding for the enzymatic activities of the carotenoid pathway required for the synthesis of zeaxanthin, but not lutein, are conserved (Figure 2). Lutein is associated with the dissipation of excess energy from the photosystems and zeaxanthin is part of the xanthophyll cycle, which has the same function (Niyogi et al., 1997). However, the loss of violaxanthin de-epoxidase shows loss of the xanthophyll cycle in these species. The fact that zeaxanthin is also a precursor of abscisic acid may explain the conservation of a functional synthesis pathway. Thus, the switch to mycoheterotrophy appears to have trimmed the multifunctional carotenoid synthesis pathway to keep only the enzymes required for its non-photosynthetic functions.

Because of the potential photo-toxicity of chlorophylls and their precursors (Rebeiz et al., 1984), a null expectation might be that mycoheterotrophic species should lose the chlorophyll synthesis pathway. It is nonetheless mostly conserved, even if incomplete, in *E. aphyllum* and *G. elata* (Figure 2). Such conservation has been observed in holoparasitic and mycoheterotrophic plants (Wickett et al., 2011; Barrett et al., 2014) and in coral-infecting apicomplexan (Kwong et al., 2019), and suggests that chlorophylls or their intermediates should have a non-photosynthetic function. It remains unclear what this function is (Ankele et al., 2007). *N. nidus-avis* differs from the two other species in having a complete and functional chlorophyll synthesis pathway. Its activity, along with other plastid activities, was detected in *N. nidus-avis*, mostly in the flowers (Figure 3). This is consistent with the detection of chlorophyll a and b in the inflorescence (Pfeifhofer, 1989). Chlorophyll was also detected in other MH orchids (Barrett et al., 2014) and the authors proposed that it would support a minimal and localized photosynthetic activity providing additional carbon to the production of seeds. This hypothesis is consistent with the demonstration that the products of the photosynthesis of the mixotrophic orchid *Cephalanthera damasonium* are targeted to fruits and seeds (Lallemand et al., 2019a). It is also supported by Menke and Schmid (1976), which reported cyclic photophosphorylation in the flower of *N. nidus-avis*. However this report is incompatible with the absence of most plastid and nuclear genes coding for photosystem I and cytochrome b6/f and deserves further study.

As free chlorophylls are photo-toxic (Rebeiz et al., 1984), the accumulation of chlorophyll requires a photo-protection mechanism. Flowers of *N. nidus-avis* are not green, but they turn green upon heating (Supplementary Figure 1), suggesting that the chlorophyll is stored in a heat-labile complex and that this may limit toxicity. Indeed, Cameron et al. (2009) failed to detect any chlorophyll fluorescence in this species, supporting its lack of photochemical activity. When compared with *G. elata* and *E. aphyllum*, the activity of the chlorophyll synthesis pathway in *N. nidus-avis* is associated with the presence of several SEP and ELIP genes. The SEP1 and ELIP Arabidopsis orthologs are induced in response to high light and are believed to bind chlorophyll (Adamska et al., 1999; Heddad, 2000; Rossini et al., 2006), but their exact molecular functions are unknown. Their conservation in *N. nidus-avis*, but not in *E. aphyllum* or *G. elata*, suggests that they may indeed bind chlorophyll to inactivate its ability to capture light.

Another, non-exclusive possible explanation for conservation of a functional chlorophyll synthesis pathway and the accumulation of zeaxanthin to high levels in *N. nidus-avis* (Pfeifhofer, 1989) may be camouflage. By visually blending the plants into the background of leaf litter, the dull colors of MH species protect them against herbivory (Klooster et al., 2009).

In any case, we show that the switch to mycoheterotrophy is mostly dominated by function losses, and does not require major, massive metabolic innovations. In mixotrophic species (representing an evolutionary transition from autotrophy to mycoheterotrophy; Selosse and Roy, 2009), a metabolomic and transcriptomic analysis showed that their response to the loss of photosynthesis by mutation was similar to the response of achlorophyllous mutants of autotrophic plants (Lallemand et al., 2019b). This suggests that the ability of achlorophyllous variants of otherwise green mixotrophic species to sustain an almost normal growth without photosynthesis is mostly based on the plasticity of plant metabolism. Furthermore, mycoheterotrophy is not a rare event (it has occurred >50 times in 17 plant families; Merckx et al., 2009; Tĕšitel et al., 2018; Barrett et al., 2019), suggesting that it primarily entails functional losses and not complex gene gains.

Another characteristic of mycoheterotrophic orchids is the lack of developed leaves. They are not missing but are reduced to small scales (Figure 1). The genes supposedly involved in leaf initiation but also leaf blade development are not missing, most probably because they function in other developmental processes. So the lack of developed leaves in mycoheterotrophic orchids could be explained by impaired expression profiles of these genes.

### An Upside-Down Metabolic Architecture

Photosynthesis is considered to be at the core of plant metabolism and so its loss in normally green plants severely impacts their metabolism (Aluru et al., 2009; Abadie et al., 2016; Lallemand et al., 2019b). We analyzed the physiology of mycoheterotrophic orchids through gene expression in different organs (Figure 3 and Supplementary Data 6). Many genes were differentially expressed, reflecting a partition of metabolic functions between the organs of most plants. The flowers showed a higher activity of cell division, primary cell wall and signaling pathways, which can be attributed to floral development. Similarly, higher phenolic compound synthesis can be associated with pollinator attraction involving flower pigmentation and production of fragrant phenolics (Jakubska-Busse et al., 2014). Conversely, the different underground organs of *N. nidus-avi*s (roots) and *E. aphyllum* (rhizome) converged toward a higher activity of pathways likely involved in the interaction with their fungal partners (microbe interactions, proteasome, and transporters). This transcriptomic convergence probably results from the similar function as organs where nutrient exchange at plant-fungus interfaces takes place. This is also evidenced in their anatomical convergence (reduced number of xylem elements) or functional similarities (nutritional independency from the other organs of plant; Rasmussen, 1995). Although *N. nidus-avis* and *E. aphyllum* showed similar pathway enrichments, especially in the aerial organs, there were some idiosyncrasies. These differences are difficult to interpret clearly as they may result from the different phylogenetic backgrounds, the anatomical differences (roots vs. rhizome) but also from different fungal partners. For example, the peak of trehalose, tryptophan, starch, and sucrose metabolism observed in the rhizome of *E. aphyllum* as opposed to a peak of tyrosine metabolism in the roots of *N. nidus-avis* (Figure 3 and Supplementary Data 6) may provide clues to the specificities of the nutrient fluxes in these two pairs of partners.

Comparing symbiotic and asymbiotic protocorms of the orchid *Serapias vomeracea*, Fochi et al. (2017) highlighted the importance of organic N metabolism and especially lysine histidine transporters (LST) in its interaction with its fungal partner. In our analysis, several LST genes were differentially expressed between the organs for both *N. nidus-avis* and *E. aphyllum*, but some were induced in flowers while others were more transcribed in stems or mycorrhizal parts (Supplementary Data 7). In a similar analysis in *G. elata*, the upregulation of clathrin genes in symbiotic protocorms compared to asymbiotic protocorms suggested the involvement of exocytosis in the interaction between the orchid and its fungal partner (Zeng et al., 2017). Our analysis showed no signal specific to N metabolism or exocytosis. The different conditions considered in these studies may help explain such discrepancies, but they may also illustrate different kinds of evolutionary adjustments occurring in different mycorrhiza.

Comparison of expression profiles of the mycoheterotrophic orchids to similar datasets in the autotrophic species: *B. distachyon* and maize provides additional evidence of the impact of mycoheterotrophy on plant metabolism. The interpretation of differences should be done carefully because it is limited by factors such as different phylogenetic backgrounds, possibly different growth conditions (including the possible absence of mycorrhizal fungi in the autotrophic plants considered here), or the restriction of the comparison to orthogroups detected in all four species. Despite these limitations, we can state that almost 40% of the analyzed orthogroups had a significantly different root/stem ratio between mycoheterotrophic and autotrophic species, and that 30% of the orthogroups, from numerous pathways, showed inverted underground organ/stem ratios, suggesting that the metabolism of mycoheterotroph species has been inverted compared to photosynthetic taxa. This inversion of the metabolism architecture likely coincided with the inversion of the usual source/sink relationship: in mycoheterotrophs, underground organs are sources, while they are a sink in photosynthetic species. The sink organs were associated with a higher activity of several major metabolic pathways (carbohydrate and nucleotide metabolism, amino acid and fatty acid biosynthesis, glycolysis, and respiration). In association with a higher DNA replication and primary cell wall activity (which involves glycosidases) and a higher expression of auxin transporters, sink organs likely experience stronger growth than their source counterparts. Mycoheterotrophic roots and rhizomes are generally short, thick and compact to minimize accidental loss of a part of a source organ and nutrient transfer effort (Imhof et al., 2013), stems are ephemeral (<2 months) but fast growing (e.g., 4 cm/day in *E. aphyllum*, J. Minasiewicz personal observations) organs involved in sexual reproduction but without nutritional functions. Conversely, fibrous roots of grasses have high growth rate as nutrient uptake depends largely on the root length (Fitter, 2002). Even with different growth habits, some pathways showed similar overall expression underground organ/stem ratios in mycoheterotrophic orchids and photosynthetic grasses. Plastid-related pathways (chlorophyll synthesis, plastid translation) are more active in stems than in underground organs, while symbiosis and trehalose degradation are more active in underground organs than stems. Trehalose is almost absent from vascular plants, where its 6-phosphaste precursor is an important growth regulator (Lunn et al., 2014). However, it is an abundant storage carbohydrate in mycorrhizal fungi and it has been suggested that it is transferred to mycoheterotrophic orchids to be cleaved into glucose (Müller and Dulieu, 1998). A comparison between leaves of achlorophyllous mutants (thus with mycohetertrophic nutrition) and green individuals in mixotrophic orchids showed an upregulation of trehalase, but also of trehalose-6-P phosphatases (TPP) and trehalose-6-P synthase (TPS; Lallemand et al., 2019b). Similarly, the mycoheterotrophic orchids demonstrated a higher underground organ/stem ratio of trehalase and TPP expression (but not TPS) compared to photosynthetic grasses. This result supports the hypothesis that trehalose is transferred from mycorrhizal fungi to mycoheterotrophic orchids. Many other nutrients are exchanged at this interface and our analysis suggests numerous differences between the trophic types: close to half of the orthogroups involved in solute transport showed different underground organ to stem ratios between autotrophic and mycoheterotrophic species. Some SWEET (Sugar Will Eventually be ExporTed) transporters were induced in the mycorrhiza of achlorophyllous MH mutants of the mixotrophic orchid *Epipactis helleborine* (Suetsugu et al., 2017) and in the protocorms of *Serapias* (Perotto et al., 2014). The three SWEET orthogroups in our analysis behaved differently between autotrophic and mycoheterotrophic species, but showed contrasting differences, indicating that autotrophic and mycoheterotrophic species both used SWEET transporters in underground organs and stems but corresponding to different orthologs. Similarly, 13 out of the 15 ABCG transporter orthogroups or 10 out of the 13 NRT1/PTR transporter orthogroups showed contrasted differences between autotrophic and mycoheterotrophic species. The same could be observed for all transporter families (Supplementary Data 7): autotrophic and mycoheterotrophic species use different orthologs for the transport of solutes in stem and roots, demonstrating extensive expression reprogramming. These differences are probably associated with changes in the fluxes of nutrients in autotrophic and mycoheterotrophic species, including in mycorrhizas. Understanding these changes is a central question in the study of mycoheterotrophy. However, the specificity of transporters can vary even within a gene family. For example, transporters of the NRT1/PTR family were identified as nitrate transporters, but some transport other molecules (Corratgé-Faillie and Lacombe, 2017). Further investigations of the changes of nutrient fluxes associated with this reprogramming of transporter expression should be directed at a detailed analysis of each orthogroup (assuming that the substrate specificity is the same for all transporters within an orthogroup). However, such an analysis should not replace direct measurement of these fluxes with labeling experiments, which will also be required to better understand the processes involved.

## Conclusion

The shift to mycoheterotrophy induces diverse changes in the genome of MH plants. From the analysis of the gene repertoires, we were not able to identify new functions associated with mycoheterotrophy, and large losses appeared to be restricted to genes exclusively involved in photosynthetic functions. This could superficially suggest that no metabolic innovation is required for mycoheterotrophy. However, our transcriptome analysis shows extensive changes in numerous pathways, probably associated with changes in the plant lifecycle and in the interaction with fungal partners induced by mycoheterotrophy. This reprogramming illustrates the versatility of plant metabolism and can be considered as a metabolic innovation in itself. It may also help explain why the shift to mycoheterotrophic nutrition has occurred so frequently in plant evolution: becoming mycoheterotrophic may be based more on reprogramming of existing metabolism and gene loss than on genetic innovation involving new genes or pathways.

## Materials and Methods

### Sampling Procedures

The specimens of *N. nidus-avis* and *E. aphyllum* were sampled in their natural habitats in southern Poland in 2017 at the peak of their flowering season, at 10.00 AM local time (Supplementary Table 1; these two species cannot be cultivated *ex situ*). Two plants per species were selected as biological replicates based on their size and healthy condition, keeping the parameters similar among the replicates. Each plant was excavated with surrounding soil. A fully open flower and associated stem were cut off and processed (see below) right away, while the underground organs were first cleaned thoroughly by gentle scrubbing and rinsing in distilled water to remove all visible soil and foreign material. In-field processing consisted of slicing and dividing material into samples of 150 mg in weight before immediate preservation in liquid nitrogen to inhibit RNA degradation. The presence of mycorrhizal fungus was checked on thin cross-sections of colonized organs adjacent to the sampling and examined later under a light microscope.

### RNA Extraction and Purification

The samples collected *in situ* were transferred from liquid nitrogen to a −80°C freezer until the RNA extraction step. Flower samples were homogenized in liquid nitrogen using TissueLyser II (Qiagen) in 2 mL Eppendorf tubes containing ceramic beads. This method has proven ineffective in processing root, rhizome, and stem tissue samples due to their hardness when frozen and so manual grinding in mortars with liquid nitrogen had to be applied instead. Homogenized material was subjected to the NucleoZol (Macherey-Nagel, Dueren, Germany) reagent extraction process following the manufacturer’s protocol with the addition of polyvinylpolypyrrolidone (PVPP) during the grinding of root and rhizome. RNAs were further purified using Agencourt RNAClean XP (Beckman Coulter, Brea, CA, United States) magnetic beads following the manufacturer’s instructions.

Digestion with DNase Max (Qiagen, Hilden, Germany) was subsequently conducted to purify RNA isolates from remaining genomic DNA contamination.

Finally, RNA integrity and purity were assessed by Agilent BioAnalyzer 2100 survey using the Plant RNA Nano Chip (Agilent Technology, Santa Clara, CA, United States) and RNA concentration was measured by RiboGreen assay (Thermo Fisher Scientific, Waltham, MA, United States). Samples exhibiting high integrity (RIN > 7) were selected for sequencing.

### Sequencing

The sequencing libraries were constructed using TruSeq Stranded Total RNA with the Ribo-Zero Plant kit (Illumina, San Diego, CA, United States), following the manufacturer’s instructions. Next, they were sequenced on a NextSeq500 (Illumina, San Diego, CA, United States) platform in a paired-end mode with read length of 150 bases. The sequences obtained were quality-controlled and trimmed using the Trimmomatic software (version 0.36, parameters PE and ILLUMINACLIP:TruSeq3-PE.fa:2:30:10:2:true LEADING:21 TRAILING:21 MINLEN:30) (Bolger et al., 2014) and the residual ribosomal RNAs were filtered out with SortMeRNA (version 2.1, against the databases silva-bac-16s-id90, silva-bac-23s-id98, silva-euk-18s-id95, and silva-euk-28s-id98 with the parameters – e 1e-07 –paired_in).

### Transcriptome Assembly

Due to the lack of reference genomes of the sampled plants, their transcriptomes were assembled *de novo* using the Trinity RNA-seq assembler (Haas et al., 2013) version v2.6.6 with all parameters at their default settings except –SS_lib_type RF. Taking into account the possible contamination of our samples collected *in situ*, which may vary between collected tissues, in order to avoid mis-assemblies and/or chimeric transcript generation, we assembled the transcriptomes of each plant tissue separately. Subsequently, the assemblies for each species were merged and the redundancy of the isoforms was decreased with the tr2aacds pipeline from the EvidentialGene package^1^ (version 2017.12.21; Gilbert, 2019). According to the pipeline description, we kept only those contigs that were classified as “main” or “noclass”, i.e., primary transcripts with alternates or with no alternates, respectively, to form the final unigene set.

### Identification of Contaminating Contigs

As our samples were collected in the field, the total RNAs contain transcripts from the microbiota associated with our plants, and especially abundant transcripts of the mycorrhizal fungi in underground organs, which means that the previous unigene set is contaminated with sequences that do not belong to the plants.

To identify and filter out these contigs, each reduced transcriptome was searched with the blastx algorithm against the NCBI NR database using Diamond software (Buchfink et al., 2015). Local Diamond version 0.9.16 installation was run with the following set of parameters: –sensitive, –index-chunks 2, –block-size 20, –max-target-seqs 50, –no-self-hits, –evalue 0.001, –taxonmap prot.accession2taxid.gz, with the latest parameter specifying the taxonomic information obtained from the NCBI ftp pages^2^. Both the NCBI NR database and the taxonomy information were current as of December 2018. All contigs with best hits inside the *Streptophyta* clade of plants were considered as *bona fide* orchid contigs.

However, this analysis may miss genes highly conserved across kingdoms. Hence, we performed an orthology analysis against several orchid and monocotyledon species. The analysis included proteomes of *N. nidus-avis* and *E. aphyllum* generated here, as well as published reference sets of *B. distachyon* (L.) P.Beauv., *Z. mays* L., *O. sativa*, and the orchids *G. elata, D. catenatum* Lindley, *A. shenzhenica* Z.J.Liu & L.J.Chen, and *P. equestris* (Schauer) Rchb.f. (see Supplementary Table 2). We identified orthologous groups using the OrthoFinder software (version 2.2.7, default parameters, except -S diamond) (Emms and Kelly, 2019).

Contigs sharing the same orthogroup as any protein of these seven species were considered as *bona fide* orchid contigs. For contigs with no hit at all we applied a further filtering criterion based on the expression pattern, i.e., we required such transcripts to be expressed in at least two out of our six samples. Expression analyses were performed with Seal from the BBTools package^3^ (version 38.22).

### Identification of the Fungal Partners

The contig sets were searched for ITS sequences using ITSx software (version 1.1.2; Bengtsson-Palme et al., 2013) and the identified contigs were queried against the UNITE database version 8.2 (Nilsson et al., 2019).

### Annotations

Annotation of transcripts was performed with the Trinotate suite^4^ (version 3.1.1; Bryant et al., 2017). Trinotate was fed with results of several independent analyses. To annotate protein domains, hmmscan from the HMMER 3.1b2 package (Eddy, 2011) was run against the Pfam-A entries from the PFAM database (Finn et al., 2016). The UniProt/SwissProt protein database was searched with blastp (Altschul et al., 1997) from the blast+ 2.7.1 package to retrieve gene ontology (GO), KEGG (Kyoto Encyclopedia of Genes and Genomes), and eggNOG annotations. The presence of signal peptides was assessed with signalP (Petersen et al., 2011) software.

Additionally, the transcripts were assigned to KEGG orthologs and pathways using the KAAS server (Moriya et al., 2007) with BLAST and the BBH (bi-directional best hit) method. They were also assigned to the Mapman4 pathways using the Mercator4 v2.0 online tool (Schwacke et al., 2019).

In all the above analyses, transcripts were represented by either their nucleotide sequences derived directly from the assembly or by their amino acid sequences, as derived from the open reading frames (ORFs) determined by the tr2aacds pipeline. To reduce technical bias when comparing species, the gene sets of all species were re-annotated with the same tools and parameters. The annotation of the orthogroups was derived from the annotations of their genes independently of the origin of these genes. Orthogroups were annotated with terms representing at least 25% of their genes.

### Comparison of Gene Sets

The quality and completeness of the final transcriptomes (unigene sets) for *E. aphyllum* and *N. nidus-avis* were benchmarked with BUSCO v3.0.2 (Seppey et al., 2019) against the *Liliopsida*:odb10 plant-specific reference database and compared with the abovementioned species. We also compared the representation of the KEGG pathways and Mapman4 bins in each species. The unigene sets of *E. aphyllum* and *N. nidus-avis* were first completed with their plastid gene lists extracted from the NCBI accessions NC_026449.1 and NC_016471.1, respectively. We counted whether a KEGG ortholog or its Mapman equivalent was detected independently of the number of genes associated with it. Fisher’s exact test was performed to compare *E. aphyllum* and *N. nidus-avis* to *G. elata* and to compare these three mycoheterotrophic orchids to the three autotrophic orchids in each pathway or bin. Pathways or bins with an adjusted *p*-value (Bonferroni adjustment) below 0.05 were considered as differentially represented.

### Gene Expression Analyses

Sequencing read libraries were mapped separately to their corresponding final transcriptome (unigene set) using BBmap (see text footnote 3). The software was run with the additional “rpkm” parameter, which yields per-contig raw counts directly along the standard SAM/BAM output files. Next, a raw count matrix was generated for each species’ unigene set and fed into edgeR (Robinson et al., 2010) for differential expression testing by fitting a negative binomial generalized log-linear model (GLM) including a tissue factor and a replicate factor to the TMM-normalized read counts for each unigene. Unigenes detected in less than three of the six samples were considered as poorly expressed and filtered out from the analysis. We performed pairwise comparisons of tissues, i.e., flower vs. underground organ (FL vs. MR), flower vs. stem (FL vs. ST), and underground organ vs. stem (MR vs. ST). The distribution of the resulting *p*-values followed the quality criterion described by Rigaill et al. (2018). Genes with an adjusted *p*-value [FDR, Benjamini and Hochberg (1995)] below 0.05 were considered to be differentially expressed.

Given the sets of up- and down-regulated genes for each species from pairwise tissue comparisons, we performed enrichment analysis for GO terms, KEGG, and Mapman4 pathways using hypergeometric tests. Terms with an adjusted *p*-value (Bonferroni adjustment) below 0.05 were considered to be enriched.

### Comparison of Underground Organ/Stem Expression Profiles Between Autotrophs and Mycoheterotrophs

Biological replicates are required to perform a statistical analysis and identify differentially expressed genes. Another constraint of this analysis was the comparison of the transcriptomes from different species. One option is to perform the same analysis as previously for each of the four species and compare the results of the enrichment analyses. However, this would lead only to very broad results at the level of pathways. The other option is to directly compare the four transcriptomes of the four species but this introduces various challenges and biases (Dunn et al., 2013). The first one is to identify the quadruplets of orthologous genes. In this study, we used the expression of the 18,259 orthogroups identified above as a proxy of the expression of the various molecular functions present in the stem and underground organs. This approximation should be taken into account when interpreting the results but is similar to the approach of McWhite et al. (2020). The second one is that the absolute read counts of each species for a given orthogroup cannot be directly compared because the number and length of the genes in each orthogroup can differ from one species to another. To remove this bias, we instead considered the underground organ/stem expression ratios.

As no equivalent dataset is available for autotrophic orchids, we used datasets from *Z. mays* and *B. distachyon* as autotrophic species for comparison. We focused on the underground and stem tissues using roots and internodes as the corresponding tissues for autotrophic monocotyledons. Expression values for *Z. mays* were extracted from the SRA project PRJNA217053. The samples SRR957475 and SRR957476 correspond to internodes, SRR957460 and SRR957461 to roots. Expression values for *B. distachyon* were extracted from the SRA project PRJNA419776. The samples SRR6322422 and SRR6322429 correspond to internodes, SRR6322386 and SRR6322417 to roots. Counts were calculated after mapping of the reads to their corresponding reference transcriptome (Zea_mays.B73_RefGen_v4.cdna.all.fa and Brachypodium_distachyon.Brachypodium_distachyon _v3.0.cdna.all.fa) using BBmap with the same parameters as previously.

Any orthogroup whose expression was not detected in at least one sample of all four species was filtered out from further analysis. As an orthogroup can group different numbers of genes from each species, the absolute counts cannot be compared directly. However, as the stem and underground organ samples are paired, it is possible to compare the underground organ/stem ratios. After normalization with the TMM method (Robinson et al., 2010) to correct the library size effect, the counts were transformed with the vst method of the coseq package v1.2 (Rau and Maugis-Rabusseau, 2018). The log2 root/shoot ratios calculated from the transformed counts were analyzed using the lmFit contrasts.fit and eBayes functions of the limma package v3.34.9 (Smyth, 2004). In our model, the log2 ratio was expressed as a linear combination of a species effect and the *p*-values corresponding to the difference between the average of the two mycoheterotrophic species and the average of the two autotrophic species were calculated. The distribution of the resulting *p*-values followed the quality criterion described by Rigaill et al. (2018). The Benjamini–Hochberg correction was used to control false discovery rate. We considered orthogroups with an adjusted *p*-value < 0.05 to have a different underground organ/stem/ratio between the mycoheterotrophic orchids and the photosynthetic grasses. Enrichment analyses were performed as described previously with orthogroups being annotated with terms representing at least 25% of their genes.

## Data Availability Statement

The reads are available at the NCBI database under Bioproject PRJNA633477. The GFF file and annotation of the unigene sets for *E. aphyllum* and *N. nidus-avis* as well as the raw count matrices are available at https://doi.org/10.15454/HR9KUX.

## Author Contributions

M-AS and ED designed the study. M-AS supervised the project. ED, MM, and MJ analyzed the data. ED, JM, and MJ wrote the manuscript. JC generated the RNA-seq data. JM, MJ, MM, and M-AS collected the samples. ED agreed to serve as the author responsible for contact and ensures communication. All authors contributed to the article and approved the submitted version.

## Conflict of Interest

The authors declare that the research was conducted in the absence of any commercial or financial relationships that could be construed as a potential conflict of interest.

## Footnotes

1 http://arthropods.eugenes.org/EvidentialGene/trassembly.html

2 ftp://ftp.ncbi.nlm.nih.gov/pub/taxonomy/

3 https://jgi.doe.gov/data-and-tools/bbtools/

4 https://trinotate.github.io/

## Supplementary Material

Supplementary Figure 1 The effect of heat on the flowers of *N. nidus-avis*.

Supplementary Table 1 Details of sampling location and dates for the studied orchids.

Supplementary Table 2 Genomic datasets used in this study.

Supplementary Table 3 Comparison of the intermediate and final assemblies generated.

Supplementary Table 4 Composition of contamination sources among sampled tissues.

Supplementary Table 5 Annotation statistics of the generated transcriptome assemblies.

Supplementary Table 6 Summary statistics of the BUSCO analysis of completeness for the generated transcriptomes in comparison to the *E. aphyllum* transcriptome from Schelkunov et al. (2018) and another mycoheterotrophic orchid *G. elata* with a sequenced genome.

Supplementary Table 7 Statistics of per-tissue read mapping to the intermediate and final assemblies.

Supplementary Table 8 Per-species statistics among the generated orthologous groups.

Supplementary Table 9 Species overlaps among orthologous groups.

Supplementary Data 1 Distribution of GO terms in the three mycoheterotrophic orchids. Only the 20 most abundant terms for each species and each ontology are shown.

Supplementary Data 2 Comparison of ortholog numbers in Mapman and KEGG pathways for the three mycoheterotrophic orchids and three autotrophic orchids. This excel file includes one sheet for each annotation plus a legend sheet.

Supplementary Data 3 Output of the Orthofinder analysis. This a tabulated file where each line corresponds to an orthogroup and each column provides the list of proteins (comma separated, using their standard ID) included in the orthogroup for a given species.

Supplementary Data 4 Composition and annotation of the mycoheterotroph-specific orthogroups. This excel file is composed of one sheet and a legend sheet.

Supplementary Data 5 Differential expression analysis of *N. nidus-avis* and *E. aphyllum* organs. This excel file includes four sheets plus a legend sheet.

Supplementary Data 6 Mapman, KEGG, and GO enrichment analysis of *N. nidus-avis* and *E. aphyllum* expression. This excel file contains six sheets and a legend sheet.

Supplementary Data 7 Differential analysis of the root/shoot expression ratios. This excel file contains two sheets and a legend sheet.

Supplementary Data 8 Mapman, KEGG, and GO enrichment analysis of the expression ratios.
