# Supplementary material for: The Genomic Impact of Mycoheterotrophy in Orchids
Source: Front Plant Sci. 2021 Jun 9;12:632033. doi: 10.3389/fpls.2021.632033 (PMC8220222; doi:10.3389/fpls.2021.632033)
Supplement: Supplementary Figure 1 — The effect of heat on the flowers of N. nidus-avis. [file Data_Sheet_1.zip › Supplementary Tables.docx]

**Supplemental Table 1:** Details of sampling dates and locations for the studied orchids.

| **Species** | **Location** | **Sampling date** |
| --- | --- | --- |
| *Neottia nidus-avis* | Tunel | 30.05.2017 |
| *Epipogium aphyllum* | Dolina Chochołowska | 21.07.2017 |

**Supplemental Table 2**: Genomic datasets used in this study.

| **Species** | **Proteome file** | **URL** |
| --- | --- | --- |
| *A. shenzhenica* | GCA_002786265.1_ASM278626v1_protein.faa | https://www.ncbi.nlm.nih.gov/genome/?term=txid1088818[orgn] |
| *G. elata* | GWHAAEX00000000.Protein.faa | https://bigd.big.ac.cn/gwh/Assembly/129/show |
| *D. catenatum* | GCF_001605985.2_ASM160598v2_protein.faa | https://www.ncbi.nlm.nih.gov/genome/?term=txid906689[orgn] |
| *P. equestris* | GCF_001263595.1_ASM126359v1_protein.faa | https://www.ncbi.nlm.nih.gov/genome/?term=txid78828[orgn] |
| *B. distachyon* | GCF_000005505.3_Brachypodium_distachyon_v3.0_protein.faa | https://www.ncbi.nlm.nih.gov/genome/?term=txid15368[orgn] |
| *O. sativa* | GCF_000005425.2_Build_4.0_protein.faa | https://www.ncbi.nlm.nih.gov/genome/10 |
| *Z. mays* | GCF_000005005.2_B73_RefGen_v4_protein.faa | https://www.ncbi.nlm.nih.gov/genome/?term=txid4577[orgn] |

**Supplemental Table 3:** Comparison of the intermediate and the final assemblies generated. Number of contigs

| **Species** | **Tissue** | **Original assembly** | **Reduced assembly (tr2aacds)** | **Decontaminated (and filtered) assembly** |
| --- | --- | --- | --- | --- |
| *Neottia nidus-avis* | Flower  Stem  Mycorrhiza | 73 798  92 780  111 698 | 65 794 | 43 451 |
| *Epipogium aphyllum* | Flower  Stem  Mycorrhiza | 50 823  61 378  162 618 | 72 437 | 38 488 |

**Supplemental Table 4:** Composition of contamination sources among sampled tissues.

|  | **Tissue** | **non-plant^*^**  **all** | **non-plant:**  **fungi** | **non-plant: metazoa** | **non-plant: other^#^** |
| --- | --- | --- | --- | --- | --- |
| *Neottia nidus-avis* | Flower  Stem  Mycorrhiza | 4 379  1 832  38 953 | 38.39% (1 681)  22.93% (420)  99.34% (38 696) | 45.51% (1 993)  72.16% (1 322)  0.10% (47) | 16.1% (705)  4.91% (90)  0.54% (210) |
| *Epipogium aphyllum* | Flower  Stem  Mycorrhiza | 1 317  179  61 928 | 8.28% (109)  65.36% (117)  79.96% (49 518) | 87.70% (1 155)  11.73% (21)  18.77% (11 621) | 4.02% (53)  22.91% (41)  1.27% (789) |

^#^non-Streptophyta, *other include e.g. Bacteria, Viruses, Stramenopiles

**Supplemental Table 5:** Annotation statistics of the generated transcriptome assemblies.

|  | ***Neottia nidus-avis*** | ***Epipogium aphyllum*** |
| --- | --- | --- |
| Number of contigs | 43 451 | 38 488 |
| With homology in NR (blastx match) | 22 679 | 21 801 |
| With homology in UniProt/SwissProt (blastx/blastp match) | 17 411 | 16 830 |
| With Pfam domain | 15 954 | 14 605 |
| With signal domain | 2 417 | 2 037 |
| With eggNOG hit | 12 494 | 11 112 |
| With KEGG pathway and KO (KAAS) | 5 592 (4 611 plant) | 5 043 (4126 plant) |
| With KEGG Orthologue (with KO ID assigned) | 13 217 (6 811) | 11 961 (6 478) |
| With Mapman4 pathway | 9 278 | 8 026 |
| With Gene Ontology  Biological Process  Molecular Function  Cellular Component | 15 865  12 949  13 545  13 433 | 15 193  12 713  13 087  13 219 |
| With any of the above annotation (% of all contigs) | 20 108 (46.28%) | 19 205 (49.90%) |

**Supplemental Table 6:** Summary statistics of the BUSCO analysis of completeness for the generated transcriptomes in comparison to the E. aphyllum transcriptome from Schelkunov et al. (2018)* and another mycoheterotrophic orchid G. elata with a sequenced genome (Youan et al. 2018)

| **Plant dataset** | **BUSCO categories** | ***N. nidus-avis***  43 451 contigs | ***E. aphyllum***  38 488 contigs | ***E. aphyllum******  37 183 contigs | ***G. elata***  18 969  genes |
| --- | --- | --- | --- | --- | --- |
| *liliopsida_odb10:*  3278 BUSCO groups | Complete (C) | 2573 (78.5%) | 2328 (71.0%) | 1779 (54.3%) | 2399 (73.2%) |
|  | Complete and single-copy (S) | 2317 (70.7%) | 2145 (65.4%) | 1677 (51.2%) | 2254 (68.8%) |
|  | Complete and duplicated (D) | 256 (7.8%) | 183 (5.6%) | 102 (3.1%) | 145 (4.4%) |
|  | Fragmented (F) | 119 (3.6%) | 135 (4.1%) | 330 (10.1%) | 162 (4.9%) |
|  | Missing (M) | 586 (17.9%) | 815 (24.9%) | 1169 (35.6%) | 717 (21.9%) |

**Supplemental Table 7:** Statistics of per-tissue read mapping to the intermediate and final assemblies.

|  |  | **To original** | **To reduced** | **To final** |
| --- | --- | --- | --- | --- |
| *Neottia nidus-avis* | Flower  Stem  Mycorrhiza | 98.59%  99.56%  99.43% | 96.82%  96.50%  95.27% | 94.90%  96.32%  69,27% |
| *Epipogium aphyllum* | Flower  Stem  Mycorrhiza | 99.40%  99.35%  98.98% | 96.37%  96.29%  93.98% | 96.01%  96.17%  74.43% |

**Supplemental Table 8:** Per-species statistics among the generated orthologous groups.

|  | **AS** | **BD** | **DC** | **EA** | **GE** | **NNA** | **OS** | **PE** | **ZM** |
| --- | --- | --- | --- | --- | --- | --- | --- | --- | --- |
| Number of genes | 21743 | 37892 | 34389 | 39518 | 18969 | 45173 | 28555 | 29894 | 58409 |
| Number of genes in orthogroups | 17101 | 32560 | 31635 | 17557 | 14087 | 14087 | 22157 | 27822 | 45025 |
| Number of unassigned genes | 4642 | 5332 | 2754 | 21961 | 4882 | 26029 | 6398 | 2072 | 13384 |
| Percentage of genes in orthogroups | 78.7 | 85.9 | 92 | 44.4 | 74.3 | 42.4 | 77.6 | 93.1 | 77.1 |
| Percentage of unassigned genes | 21.3 | 14.1 | 8 | 55.6 | 25.7 | 57.6 | 22.4 | 6.9 | 22.9 |
| Number of orthogroups containing species | 11732 | 14530 | 12852 | 10898 | 10828 | 11999 | 14211 | 12508 | 14721 |
| Number of species-specific orthogroups | 24 | 41 | 42 | 23 | 5 | 16 | 13 | 12 | 75 |
| Number of genes in species-specific orthogroups | 155 | 236 | 169 | 134 | 44 | 133 | 58 | 35 | 482 |
| Percentage of genes in species-specific orthogroups | 0.7 | 0.6 | 0.5 | 0.3 | 0.2 | 0.3 | 0.2 | 0.1 | 0.8 |

**Supplementary Table 9:** Species overlaps among orthologous groups.

|  | **AS** | **BD** | **DC** | **EA** | **GE** | **NNA** | **OS** | **PE** | **ZM** |
| --- | --- | --- | --- | --- | --- | --- | --- | --- | --- |
| AS | **11732** | 10332 | 10953 | 9314 | 9628 | 10166 | 10070 | 10740 | 10380 |
| BD |  | **14530** | 11014 | 9246 | 9594 | 10048 | 13253 | 10792 | 13688 |
| DC |  |  | **12852** | 9886 | 10303 | 10851 | 10718 | 11972 | 11078 |
| EA |  |  |  | **10898** | 9445 | 10187 | 9083 | 9794 | 9363 |
| GE |  |  |  |  | **10828** | 10003 | 9356 | 10209 | 9642 |
| NNA |  |  |  |  |  | **11999** | 9864 | 10709 | 10176 |
| OS |  |  |  |  |  |  | **14211** | 10524 | 13198 |
| PE |  |  |  |  |  |  |  | **12508** | 10863 |
| ZM |  |  |  |  |  |  |  |  | **14721** |
