# Supplementary figures and images for: The Genomic Impact of Mycoheterotrophy in Orchids
Source: Front Plant Sci. 2021 Jun 9;12:632033. doi: 10.3389/fpls.2021.632033 (PMC8220222; doi:10.3389/fpls.2021.632033)

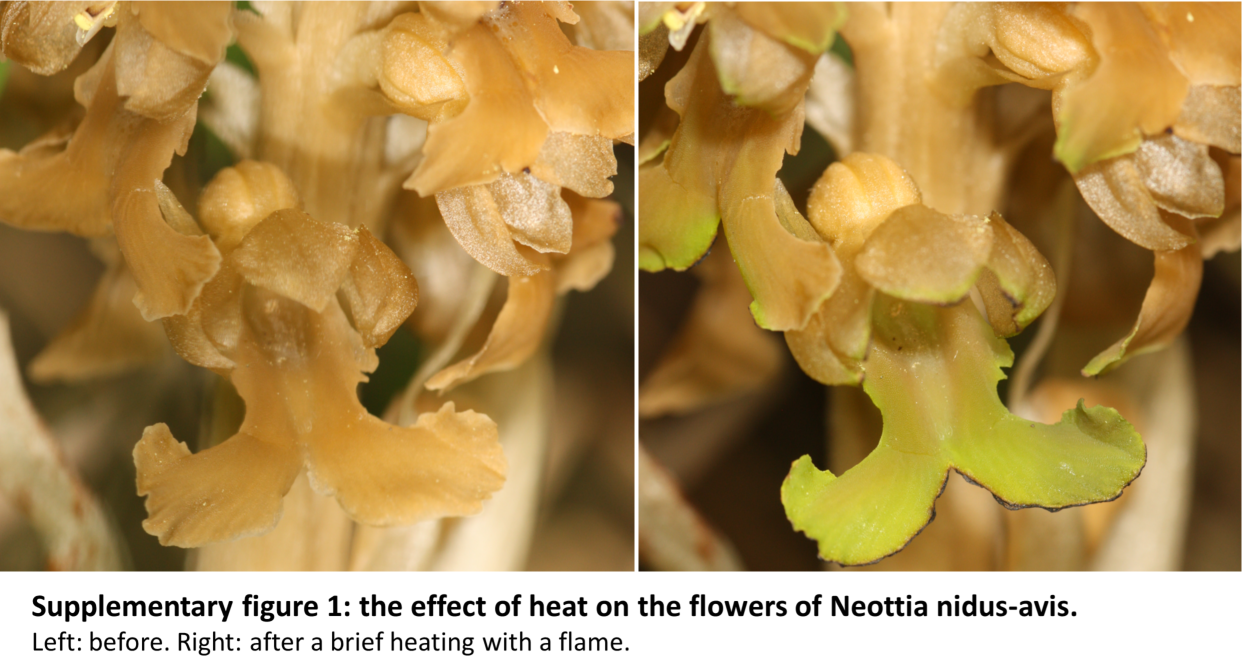

Supplement: Supplementary Figure 1 — The effect of heat on the flowers of N. nidus-avis. [file Data_Sheet_1.zip › Supplementary Figure 1.tiff]
